# Circular RNA hsa-circ-0007766 modulates the progression of Gastric Carcinoma via miR-1233-3p/*GDF15* axis

**DOI:** 10.7150/ijms.46261

**Published:** 2020-06-23

**Authors:** Weiguo Xu, Bin Zhou, Jun Wu, Pan Jiang, Huanqiu Chen, Feng Yan

**Affiliations:** 1 Department of General Surgery, The Affiliated Cancer Hospital of Nanjing Medical University & Jiangsu Cancer Hospital & Jiangsu Institute of Cancer Research, Nanjing 210009, China.; 2 Department of Clinical Laboratory, The Affiliated Brain Hospital of Nanjing Medical University, Nanjing 210009, China.; 3 Department of Clinical Laboratory, The Affiliated Cancer Hospital of Nanjing Medical University & Jiangsu Cancer Hospital & Jiangsu Institute of Cancer Research, Nanjing 210009, China.

**Keywords:** hsa-circ-0007766, miR-1233-3p, * GDF15*, gastric carcinoma

## Abstract

Circular RNAs (circRNAs), a new kind of non-coding RNAs, have gradually been proved to be critical regulators of gene expression; however, the underlying mechanisms still need to be elaborated. In the present study, we investigated the role of hsa-circ-0007766 in gastric carcinoma (GC). Quantitative real-time PCR was applied to detect the differential expression levels of circRNA, miRNAs, and mRNAs in human tissues and specific cell lines. GC cell lines were transiently transfected with siRNA. Then the proliferation, migration, and invasion assays were performed to evaluate the effect of hsa-circ-0007766 in GC cell lines. Fluorescence *in situ* hybridization, RNA pulldown assay was used to confirm the location of hsa-circ-0007766 and its relationship with miR-1233-3p. Luciferase reporter assay was then conducted to verify the interaction between miR-1233-3p and *GDF15*. Interestingly, we found that hsa-circ-0007766 was highly expressed in human GC tissues and GC cell lines. Knock-down of hsa-circ-0007766 inhibited cell proliferation, migration, invasion, and down-regulated the expression of* GDF15*. Moreover, hsa-circ-0007766 was identified as a sponge of miR-1233-3p, which could target gene *GDF15* to regulate the progression of GC. Finally, hsa-circ-0007766 was evaluated to be a valuable diagnostic marker with a sensitivity of 53.33% and specificity of 83.33% by ROC analysis. This study unveils a mechanism by which hsa-circ-0007766 regulates *GDF15* via hsa-circ-0007766/miR-1233-3p/*GDF15* axis, which may provide new insight for GC therapeutic strategies.

## Introduction

Gastric cancer is a common malignant tumor in the world, and its prognosis is relatively poor 1. According to the statistics of The International Agency for Research on Cancer (IARC) in 2012, there were about 951,000 new cases of gastric cancer in the world and about 723,000 deaths due to gastric cancer, which was ranked 5th in the incidence of malignant tumors and 3rd in mortality respectively 2. Despite significant advances in the field of individualized therapy, gastric cancer (GC) remains a clinically challenging disease due to the lack of reliable molecular tools to predict the outcomes of patients. Therefore, a better understanding of the molecular mechanisms involved in the progression of gastric cancer is significant for the development of new therapeutic strategies 3.

Circular RNAs (circRNAs), circular closed-loop RNA molecules that competitively inhibit target genes by absorbing specific miRNAs, regulate cell proliferation, differentiation, and apoptosis, and participate in tumorigenesis 4-8. They play an essential role in the growth and development of human diseases such as malignant tumors of the digestive tract, heart diseases, nervous system diseases, and diabetes 9-11. Due to its high plasma concentration, and high specificity, circRNAs have the potential to become a novel biomarker and play a vital role in the early diagnosis, treatment, and prognosis of tumors 11, 12. Besides, the recent evidence suggests that circRNAs have the function of translating proteins, which indicates that circRNAs are no longer strictly non-coding RNAs and provides a new direction for studying the role of circRNAs in tumors 7, 13. We experimentally searched the CircBase 14, CircInteractome 15, Starbase v2.0 16, and found that hsa-circ-0007766 is generated from five exons (676 bp) of erb-b2 receptor tyrosine kinase 2 (*ERBB2*, chr17:37864573-37866734). We then selected hsa-circ-0007766 as the target of our research as the emerging application of Trastuzumab in *Her2* (*ERBB2*) positive patients diagnosed with gastric carcinoma 17. To our knowledge, it is the first time to elaborate on the biological characteristics of hsa-circ-0007766 in GC.

Growth Differentiation Factor 15 (*GDF15*), a member of transforming growth factor-beta superfamily, has been used to predict disease progression in cancer, cardiovascular disease, chronic renal dysfunction, and pulmonary embolism 18-23. Also, *GDF15* was accepted as a factor to promote cell viability, invasion, migration, angiogenesis, and apoptosis in human GC cell lines 24, 25. MicroRNAs (miRNAs) are a class of single-stranded, endogenous, non-coding RNAs that play an essential role in regulating the expression of specific genes by binding to the 3'UTR of mRNA 26. As circRNAs were reported to be miRNAs sponge, we speculated that *GDF15* could be regulated by sponge effect. Therefore, we assumed that hsa-circ-0007766 might be involved in the progression of GC by regulating the expression of *GDF15* via the miR-1233-3p/*GDF15* axis.

## Materials and Methods

### Tissue samples

Totally 30 pairs of GC samples were obtained from patients diagnosed with GC who underwent gastrectomy in The Affiliated Cancer Hospital of Nanjing Medical University between 2015 and 2017. All samples were snap-frozen and stored at -80 °C until RNA or protein extraction. The study was conducted following the Declaration of Helsinki. These patients signed all informed consent, and the ethics committee has approved this study of The Affiliated Cancer Hospital of Nanjing Medical University (NYDLS-2019-919).

### Cell culture and transfection

Human gastric cancer cell lines SNU-216, HGC-27, BGC-823, SGC-7901, and the human gastric epithelial cell line GES-1 were obtained from the Type Culture Collection of the Chinese Academy of Sciences (Shanghai, China). SNU-216 and GES-1 were cultured with DMEM medium (Gibco, USA) containing 10% fetal bovine serum (FBS). BGC-823 and SGC-7901 were cultured with RPMI 1640 medium (Gibco, USA) with 10% FBS. HGC-27 was cultured with RPMI 1640 medium (Gibco, USA) with 20% FBS. The cell lines were cultured in a humidified incubator at 37 °C with 5% CO_2_. The cells were harvested by trypsinization and seeded in a 6-well plate (2 × 10^5^ cells/well). Twenty-four hours later, 100 nM of siRNAs or miRNAs were transfected into cells using Lipofectamine 2000 (Invitrogen, USA). The sequences of the siRNA and control group were demonstrated in Table 1. The detailed procedure of transfection was conducted according to the manufacturer's instructions.

### RNA extraction and quantitative real-time polymerase chain reaction (qRT-PCR)

Total RNA was extracted with TRIzol reagent (Invitrogen, USA), and cDNA was synthesized with the PrimeScript RT reagent Kit (Takara, Japan) or the Bulge-Loop miRNA Starter Kit (RiboBio, Guangzhou, China) according to the instructions. Then, qPCR was performed with SYBR Premix Ex Taq (Takara, Japan) in LightCycler 1.5 (Roche, Switzerland). PCR amplification was performed in a 20 μL reaction system including 2 μL cDNA, 6 μL DEPC, 10 μL SYBR Green Mix, 1 μL forward primer, and 1 μL reverse primer. RT reaction conditions were 60min at 42 °C and 10 min at 70 °C. PCR conditions were as follows: 95 °C for 2 min; 40 cycles of 95 °C for 15 sec and 60 °C for 60 sec. *GAPDH* served as an internal control for hsa-circ-0007766 and *GDF15*, and U6 was used as an internal control for miRNAs. The primers used in qPCR were listed in Table 1. Total RNA was incubated for 15 min at 37 °C with 3 units of RNase R (Epicentre) per 1 mg RNA. RNA was subsequently purified by phenol-chloroform extraction and reprecipitated in three volumes of ethanol. The relative expression levels were calculated using the 2^-ΔΔCt^ method. All results were expressed as mean ± SEM of three independent experiments.

### Cell proliferation assay and colony formation assay

About 2000 transfected cells were seeded into each well of 96-well plates, incubated at 37 °C with 5% CO2. CCK-8 solution (10 μL) was added to each well, and absorbance at 450 nm was detected at 0, 24, 48, 72, 96 hours after seeding with a microplate reader. Then, about 1000 transfected cells were seeded in each well of 6-well plates and cultured for ten days. Cell colonies were fixed using 4% paraformaldehyde for 20 min and stained with Giemsa solutions (KeyGen Biotech, China) for another 20 min. Colony numbers in each group were counted using ImageJ software.

### Migration and invasion assays

In this study, about 5 × 10^4^ transfected cells were suspended in 200 μL serum-free medium and seeded into the upper chamber without Matrigel for migration assay. Analogously, about 8 × 10^4^ transfected cells containing 200 μL serum-free medium were seeded into the Transwell Matrigel-coated upper chamber (Corning^®^ Matrigel^®^ invasion chamber, USA) for invasion assay. Medium with 10% FBS was added into the lower chamber as a chemoattractant. The cells were incubated for 24 hours for migration assay and 48 hours for invasion assay. We removed the cells on the top side of the chamber with cotton swabs. Then the bottoms of the chambers were fixed with 4% paraformaldehyde for 20 min and stained with 0.1% crystal violet for another 20 min. Finally, we counted the invaded cells and captured the photographs with a microscope (magnification × 100).

### CircRNA Fluorescent *in situ* Hybridization (FISH)

Cy3-labeled hsa-circ-0007766 probe and negative control were purchased from RiboBio (Guangzhou, China). A Ribo™ ncRNA FISH kit (cat. no. C10910) purchased from RiboBio (Guangzhou, China) was employed for circRNA FISH to identify the location and expression of hsa-circ-0007766 in gastric cancer cell lines (HGC-27 and SNU-216) according to the manufacturer's protocol. In brief, cells were cultured and fixed in 4% paraformaldehyde for 10 min at room temperature. Cells were permeabilized with Triton-100 for 5 min at 4 °C (Beyotime, Shanghai, China), then Cy3-labelled hsa-circ-0007766 and DAPI-labelled 18S-RNA probes (RiboBio, Guangzhou, China) were detected. In the dark, DNA was stained with DAPI for 10 min at room temperature, followed by washing in PBS three times every 5 min. Slides were mounted and examined by confocal fluorescence microscopy (×200; Olympus Corporation, Tokyo, Japan).

### Pulldown assay with Biotin-labelled circRNA probe

The biotinylated probe was synthesized by RiboBio (Guangzhou, China). The sequence of the probe was specifically designed to bind to the back-spliced junction of hsa-circ-0007766, while the scramble probe was used as the negative control. About 1 × 10^7^ cells were lysed in lysis buffer (25 mM Tris•HCl pH 7.4, 150 mM NaCl, 6 mM MgCl2, 1% NP-40, 1 mM EDTA, 5% glycerol, 1 × Proteinase Inhibitor cocktail, 60 U/mL RNase inhibitor). Cell lysates were incubated with 3 μg biotinylated probes or scramble probes for two hours in RT. Then the biotin-circRNA-RNA complex was pulled down by incubating the lysates with washed streptavidin magnetic beads (Invitrogen, USA) for another four hours in 4 °C. Finally, the beads were washed five times with lysis buffer, and RNA complexes combining on the beads were extracted with TRIzol reagent for further research.

### Pulldown assay with Biotin-labelled miRNA probe

The biotinylated miRNA mimics probe and the scramble probe were designed by RiboBio (Guangzhou, China). The sequences of probes were listed in Table 1. About 2 × 10^6^ cells were transfected with 50 μM of biotinylated miRNA mimics probe or the scramble probe at 50% confluency for lysis. The lysates were incubated with blocked streptavidin magnetic beads (Invitrogen, USA) for four hours in 4 °C. Next, the beads were washed five times, and the binding RNAs were purified with TRIzol reagent for qRT-PCR. Then we analyzed the product of PCR by agarose gel electrophoresis.

### Dual-luciferase reporter assay

GC cell lines HGC-27 and SNU-216 were cultured in 24-well plates and then cells were co-transfected with plasmids containing 3'UTR of *GDF15* and miR-1233-3p mimics using Lipofectamine 2000 (Invitrogen, USA). The *GDF15* 3′-UTR wild-type and a* GDF15* 3′-mutant were synthesized and inserted into the pmirGLO luciferase reporter vector to produce pmirGLO-*GDF15*-wt and pmirGLO-*GDF15*-mut constructs, respectively. The cells were co-transfected with wild-type or mutant-type reporter plasmid vectors and miR-1233-3p mimics or negative control. The luciferase activities of firefly and renilla were detected by the dual-luciferase reporter assay system (Promega, Massachusetts, USA). The ratios were calculated by normalizing the firefly luciferase activity with that of renilla, and each test was repeated for three independent assays.

### Western blot

We used RIPA lysis buffer (Beyotime, Shanghai, China) to extract protein from SNU-216 and HGC-27 cells. Then BCA Protein assay kit (Beyotime, Shanghai, China) was applied to detect the protein concentration. Protein extractions (100 μg) were separated by 10% SDS-PAGE and transferred onto polyvinylidene fluoride (PVDF) membranes. Afterwards, the membrane was blocked with 5% nonfat milk (BD Biosciences, USA) and 0.1% Tween 20 in tris-buffered saline. Then the blocked membrane was immunoblotted overnight using anti-*GDF15* antibody (1:1000, #A0185; ABclonal, China) and anti-beta-actin antibody (1:2000, #AC026; ABclonal, China) at 4 °C with gentle shaking. The membranes were blocked in secondary antibodies (1:5000, ABclonal, China) at room temperature for two hours, and protein bands were visualized by an ECL kit (Keygen, Nanjing, China). Band intensity was quantified using a chemiluminescent and multicolor fluorescent imaging system and normalized to the respective Actin bands. Immunoreactivity was detected with the Odyssey fluorescent scanning system (LI-COR) and analyzed by Image Studio software.

### Statistical analysis

All the analyses were performed with SPSS 19.0 (IBM, SPSS, Chicago, IL, USA) and GraphPad Prism 6, and all data were presented as mean ± standard error of the mean (SEM). Significant differences were calculated using the Student's t-test or Chi-square test. Pearson's Coefficient was conducted to analyze the pertinence of qPCR results. Software MedCalc was applied to make the receiver operating characteristic (ROC) curve and calculate the area under curve (AUC), sensitivity, and specificity. The sensitivity-specificity relationship was determined using the Youden's J index. P < 0.05 was considered to be statistically significant.

## Results

### Hsa-circ-0007766 was high-expressed in GC tissues and cell lines and was correlated with the clinical-pathological parameters

**Figure 1:**
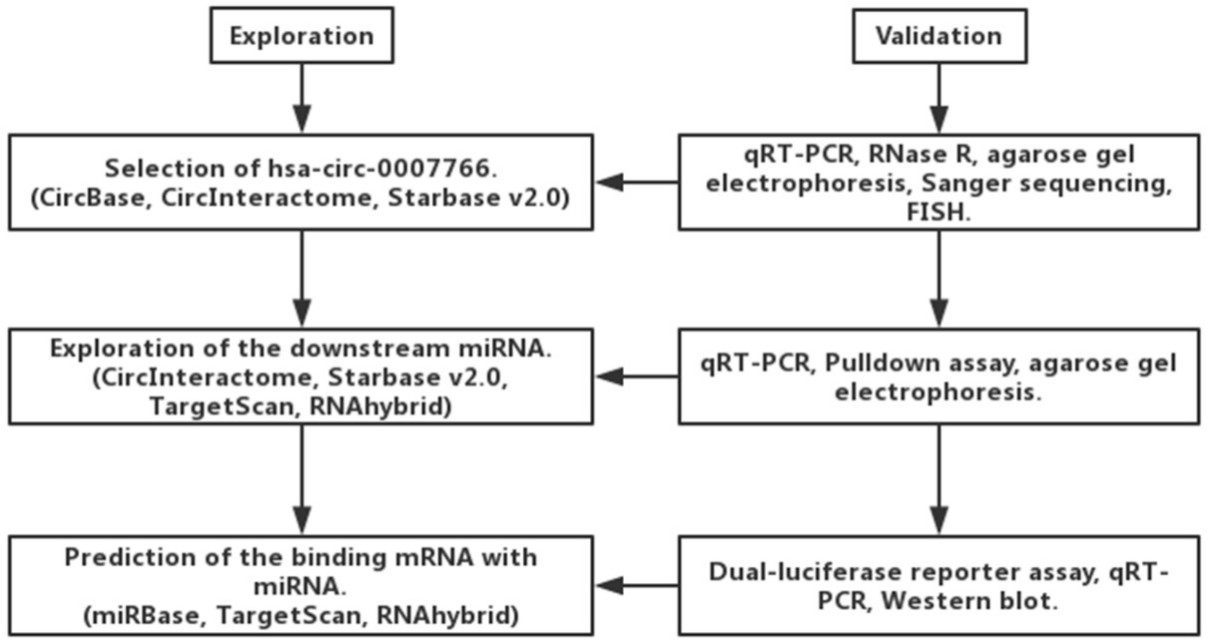
Exploration and validation of circRNA, miRNA, and mRNA based on the bioinformatic analysis.

**Figure 2:**
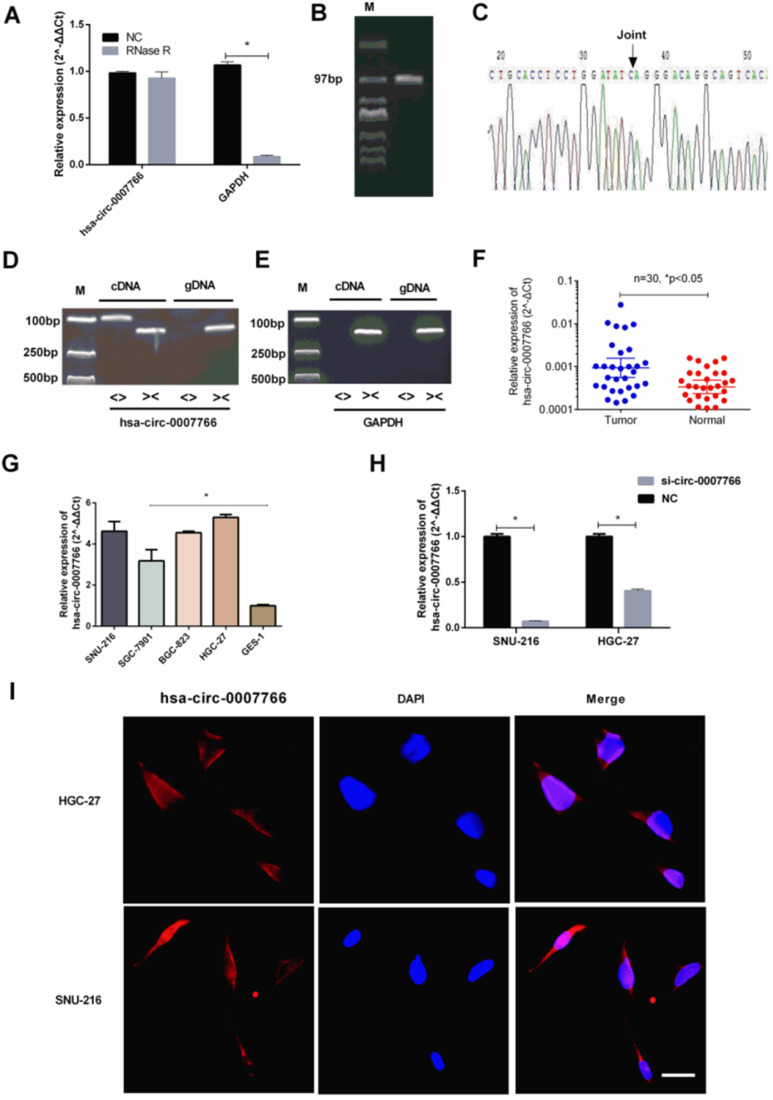
** Hsa-circ-0007766 were verified and high-expressed in GC tissues and cell lines. (A)** Total RNA was treated with RNase R. Hsa-circ-0007766 was stably expressed in both RNase R(-) and RNase R(+) total RNA samples.** (B)** The divergent primers of hsa-circ-0007766 were designed, and the qRT-PCR product was verified with Sanger sequencing. **(C)** The result of Sanger sequencing, black arrow indicated the special splicing junction of hsa-circ-0007766. **(D-E)** qRT-PCR assay with divergent or convergent primers indicating the existence of hsa-circ-0007766 in GC cell lines. GAPDH was used as a negative control.** (F)** qRT-PCR assay with divergent primers indicated the high expression of hsa-circ-0007766 in 30 pairs of human GC tissues compared with their adjacent normal tissues. **(G)** The expression of hsa-circ-0007766 in SNU-216, SGC-7901, BGC-823, HGC-27, and GES-1 cell lines was measured by qRT-PCR. **(H)** qRT-PCR analysis verified the effective down-regulation of hsa-circ-0007766 after the transfection of the siRNA for 48 hours in SNU-216 or HGC-27 cells. **(I)** FISH detection of hsa-circ-0007766 in SNU-216 and HGC-27 cells. Nuclei were stained blue with DAPI. Hsa-circ-0007766 was stained red with cy3. (Data are mean ± SEM of three experiments. The Student's t-test analyzed the difference in **F-H**, * P < 0.05 vs. Normal gastric tissues, GES-1, or negative control. Scale bar= 10 µm).

Hsa-circ-0007766 was selected by us with several databases, and it was validated by RNase R digestion and qRT-PCR (Figure 1). We observed that hsa-circ-0007766 was resistant to RNase R, whereas the linear control was not stable with RNase R treatment (Figure 2A). We then designed the divergent primers for hsa-circ-0007766 and verified the qRT-PCR product with Sanger sequencing (Figure 2B-2C). Next, the divergent and convergent primers for hsa-circ-0007766 were designed to amplify the cDNA of circRNA and gDNA of linear RNA from GC cell line HGC-27 by qRT-PCR. We confirmed that cDNA of circRNA could be amplified by both primers; however, gDNA could only be amplified by convergent primers. The results of Sanger sequencing verified the back-spliced junction, and the gel electrophoresis was applied to confirm the product of qPCR for circRNA and linear RNA (Figure 2D-2E). Then we detected 30 pairs of GC tissues and matched adjacent normal tissues with qRT-PCR to verify the expression level of hsa-circ-0007766. We found hsa-circ-0007766 was significantly high-expressed in these tissues and several GC cell lines (SNU-216, HGC-27, BGC-823, SGC-7901) compared with matched adjacent normal tissues and normal gastric epithelial cell line GES-1, respectively (Figure 2F-2G). And designed siRNA could efficiently down-regulate the expression of hsa-circ-0007766 (Figure 2H). Besides, we analyzed the results of qRT-PCR, we found that the expression level of hsa-circ-0007766 was significantly correlated with histological grade rather than other parameters (age, sex, tumor size, TNM stage) (Table 2). We then conducted the FISH assay in GC cell lines and found hsa-circ-0007766 was mainly located in the cell cytoplasm (Figure 2I).

### Down-regulated hsa-circ-0007766 inhibited the progression of GC cells *in vitro*

**Figure 3:**
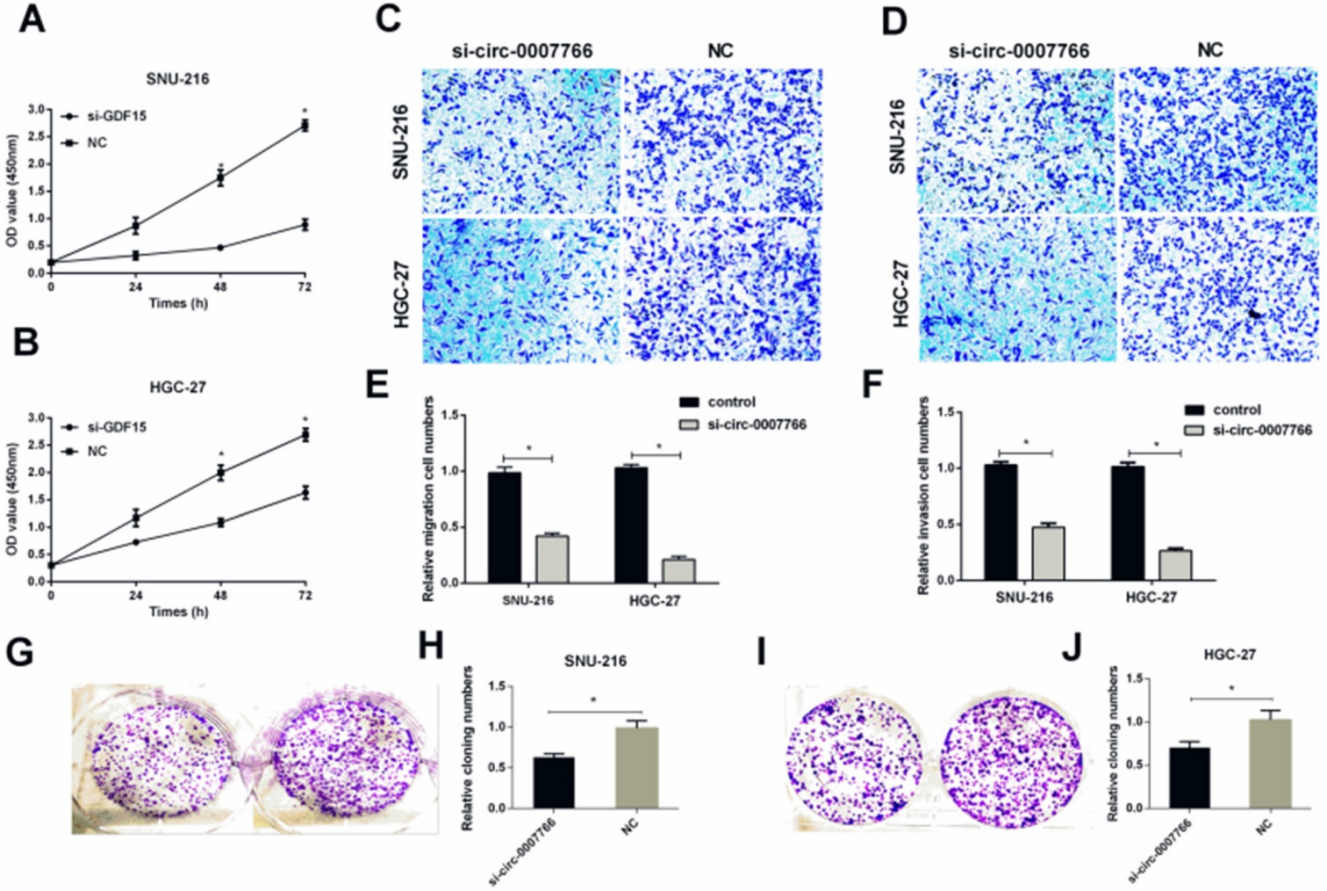
** Down-regulated hsa-circ-0007766 inhibited the progression of GC cells *in vitro*. (A)** and **(B)** Down-regulation of hsa-circ-0007766 inhibited cell proliferation as indicated by CCK-8 assays in SNU-216 and HGC-27 cells. **(C)** and** (E)** Transwell migration assays were measured, and the results were expressed as relative migration cell numbers compared with respective control (magnification, x100, *P < 0.05, Student's t-test). **(D)** and** (F)** Transwell invasion assays were measured, and the results were expressed as relative invasion cell numbers compared with respective control (magnification, x100, *P < 0.05, Student's t-test). **(G-J)** Down-regulation of hsa-circ-0007766 inhibited the colony formation of SNU-216 (**G, H**) and HGC-27 **(I, J)** cell lines as demonstrated by colony formation assays. (*P < 0.05, Student's t-test).

SiRNA or negative control (NC) was transiently transfected into SNU-216 or HGC-27 with lipofectamine 2000 to investigate the underlying mechanism of hsa-circ-0007766 in GC cell lines. We found that the expression level of hsa-circ-0007766 was down-regulated for about 10-16 folds with siRNA (Figure 2H). The CCK-8 assay suggested that down-regulated circRNA could inhibit the proliferation of SNU-216 and HGC-27 cell lines compared with the NC group obviously (Figure 3A-3B). Besides, transwell migration and invasion assays demonstrated that knockdown of hsa-circ-0007766 could also suppress the migration and invasion abilities of SNU-216 and HGC-27 cell lines (Figure 3C-3F). We then applied colony formation assays to assess the growth ability of GC cells and found that down-regulated hsa-circ-0007766 could significantly suppress the growth of SNU-216 and HGC-27 cell lines (Figure 3G-3J). The assays above showed that down-regulated hsa-circ-0007766 inhibited the progression of GC cells *in vitro*.

### Hsa-circ-0007766 acted as the miRNA sponge for miR-1233-3p

**Figure 4:**
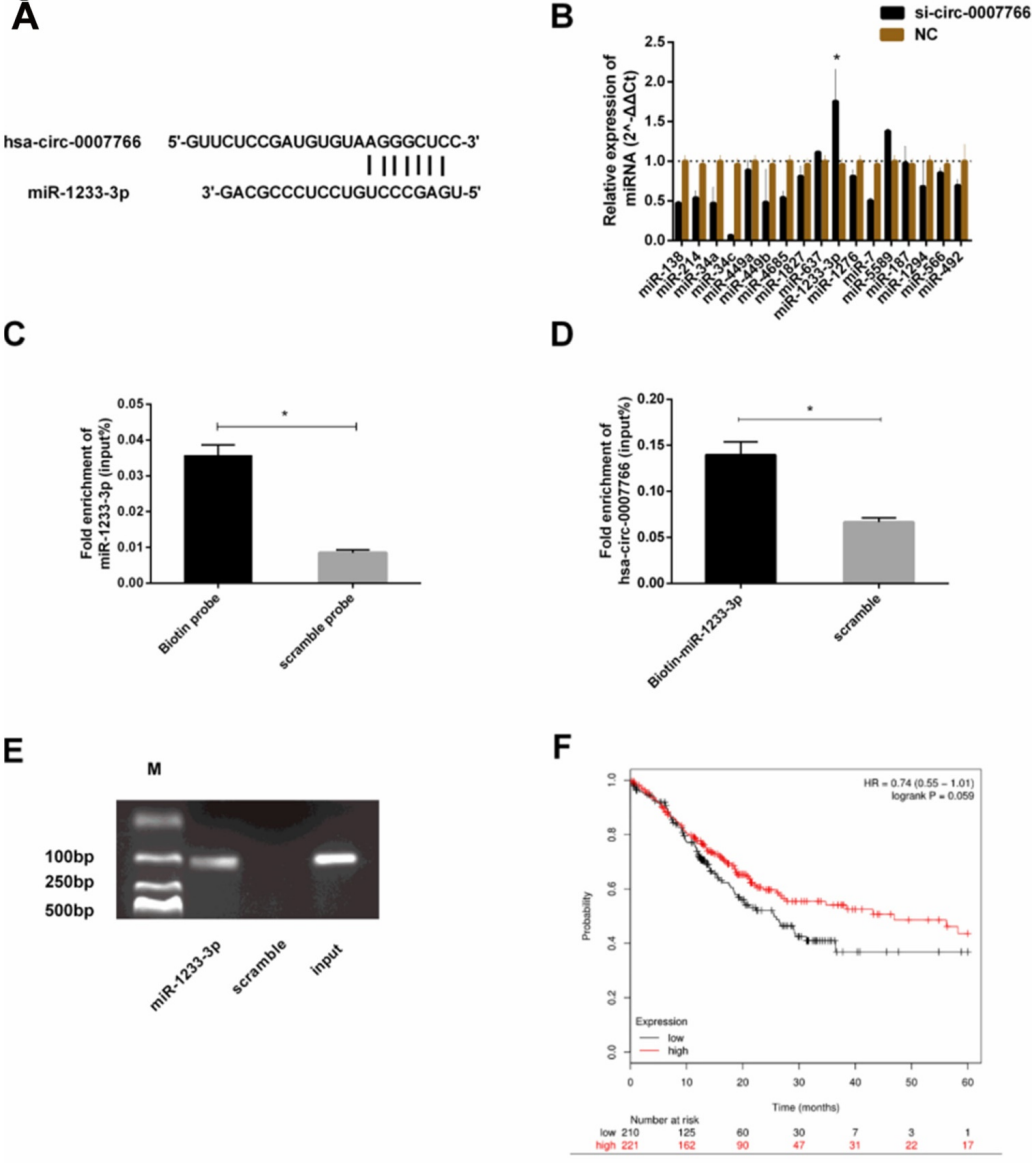
** Hsa-circ-0007766 acted as a miRNA sponge for miR-1233-3p. (A)** Bioinformatic analysis (CircInteractome, Starbase V2.0, RNAhybrid, and TargetScan) of the putative binding sites of hsa-circ-0007766 with miR-1233-3p. **(B)** qRT-PCR assay with divergent primers verified the rise of miR-1233-3p after the transfection of siRNA for hsa-circ-0007766. **(C)** MiR-1233-3p pulled down by biotin-labelled hsa-circ-0007766 could be detected by qRT-PCR significantly compared with the scramble group. **(D)** Biotin-labelled miR-1233-3p captured more hsa-circ-0007766 than the scramble group. **(E)** The product of **(D)** was detected using qRT-PCR, followed by agarose gel electrophoresis. **(F)** Kaplan-Meier plotter analysis of the correlation between miR-1233-3p expression level and the overall survival of GC patients. (*P < 0.05, Student's t-test).

To explore the downstream miRNAs, we analyzed CircInteractome 15, Starbase v2.0 16, RNAhybrid 27 and TargetScan 28 to predict the binding sites of hsa-circ-0007766 with miR-1233-3p (Figure 1, Figure 4A). We found that hsa-circ-000776 might probably bind with several miRNAs (miR-1233-3p, miR-1276, miR-136-5p, miR-187, miR-566, miR-637, miR-34a, miR-449a, miR-449b-5p, miR-377-3p, miR-34a-5p, miR-34c-5p). We attempted to figure out the expression correlation between hsa-circ-0007766 and these miRNAs by qRT-PCR, and we found that miR-1233-3p was significantly up-regulated after the knockdown of hsa-circ-0007766 (Figure 4B). Consistently, a pulldown assay with a biotin-labelled circRNA/miRNA probe further confirmed this interaction. As shown in Figure 4C, miR-1233-3p pulled down by biotin-labelled hsa-circ-0007766 could be detected by qRT-PCR compared with the scramble group. Meanwhile, biotin-labelled miR-1233-3p captured more hsa-circ-0007766 than the biotin-NC (Figure 4D-4E). These data confirmed the interaction between hsa-circ-0007766 and miR-1233-3p. Then we demonstrated the correlation between miR-1233-3p levels and the prognostic outcomes of GC patients using the Kaplan-Meier Plotter website 29. Patients were stratified into two groups based on an auto select best cut-off. We found that miR-1233-3p expression levels were correlated with survival data (Figure 4F).

### GDF15 was a downstream target of miR-1233-3p

**Figure 5:**
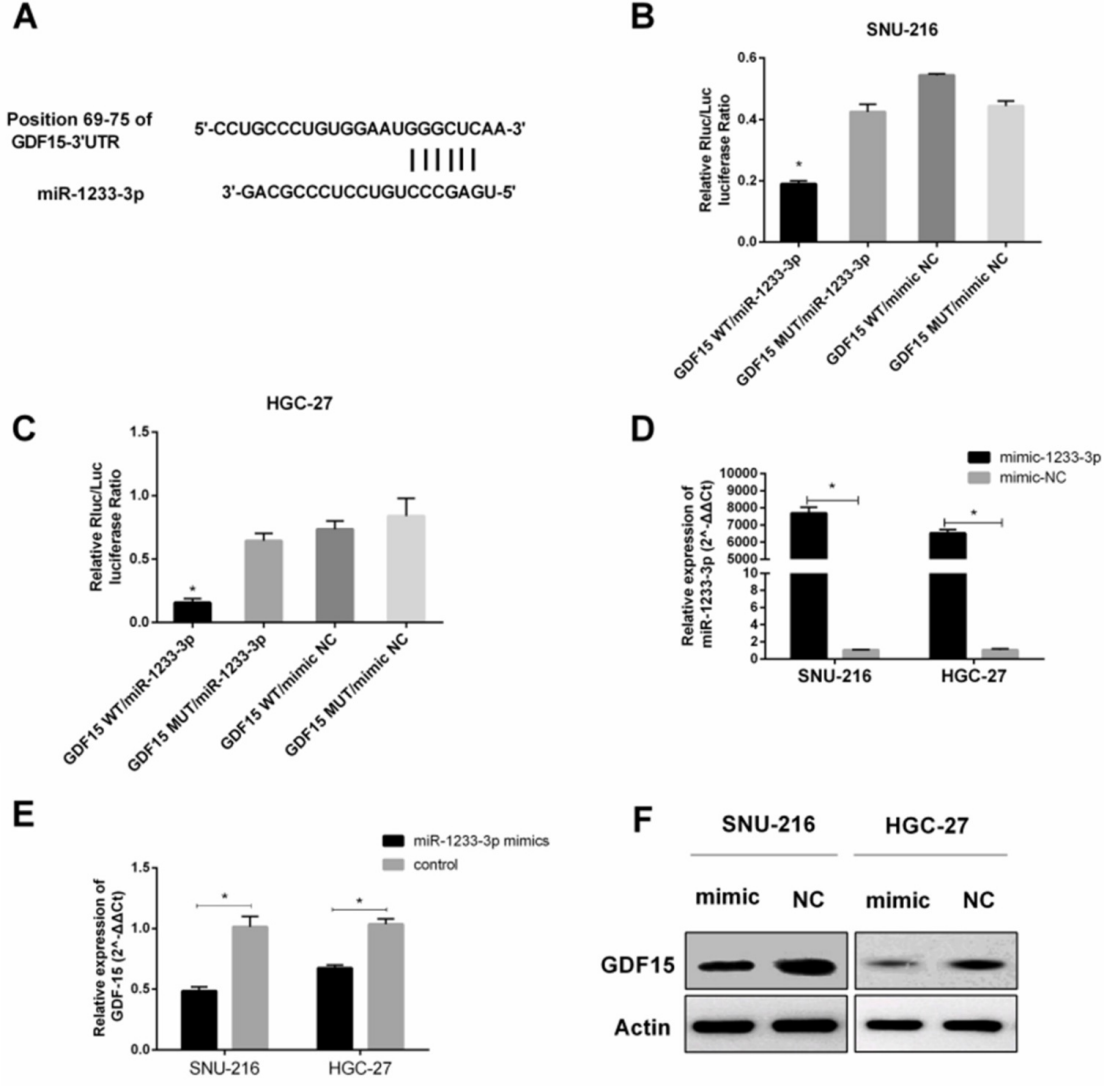
***GDF15 was* a downstream target of miR-1233-3p. (A)** Bioinformatic analysis (miRBase, Targetscan, RNAhybrid) of the putative binding sites of 3'UTR of *GDF15* with miR-1233-3p. **(B)** and** (C)** Dual-luciferase reporter assay was conducted to verify the binding between 3'UTR of *GDF15* (wild or mutant fragments) and miR-1233-3p in SNU-216 and HGC-27. We found that miR-1233-3p mimics could significantly reduce the ratios of luciferase activity. **(D)** MiR-1233-3p mimics could significantly up-regulate the expression of miR-1233-3p in SNU-216 and HGC-27. **(E)** Transfection of miR-1233-3p mimics down-regulated the expression of *GDF15* in SNU-216 and HGC-27. **(F)** Western blot assay showed that miR-1233-3p mimics could partly reduce the protein expression of *GDF15*. (*P < 0.05, Student's t-test).

We consulted related literature to find that *GDF15* might be the downstream gene of miR-1233-3p (Figure 1, Figure 5A). Then luciferase reporter assay was conducted to verify this binding between* GDF15* mRNA (3'UTR of wild or mutant fragments) and miR-1233-3p, and we found that miR-1233-3p mimics could significantly reduce the ratios of luciferase activity (Figure 5B-5C). MiR-1233-3p mimics could significantly up-regulate the expression of miR-1233-3p in SNU-216 and HGC-27 (Figure 5D). Next, miR-1233-3p mimics were transfected into GC cell lines, and we found that the expression of *GDF15* was significantly reduced by qRT-PCR or western blot (Figure 5E-5F).

### The silence of GDF15 inhibited the progression of GC cells *in vitro*

**Figure 6:**
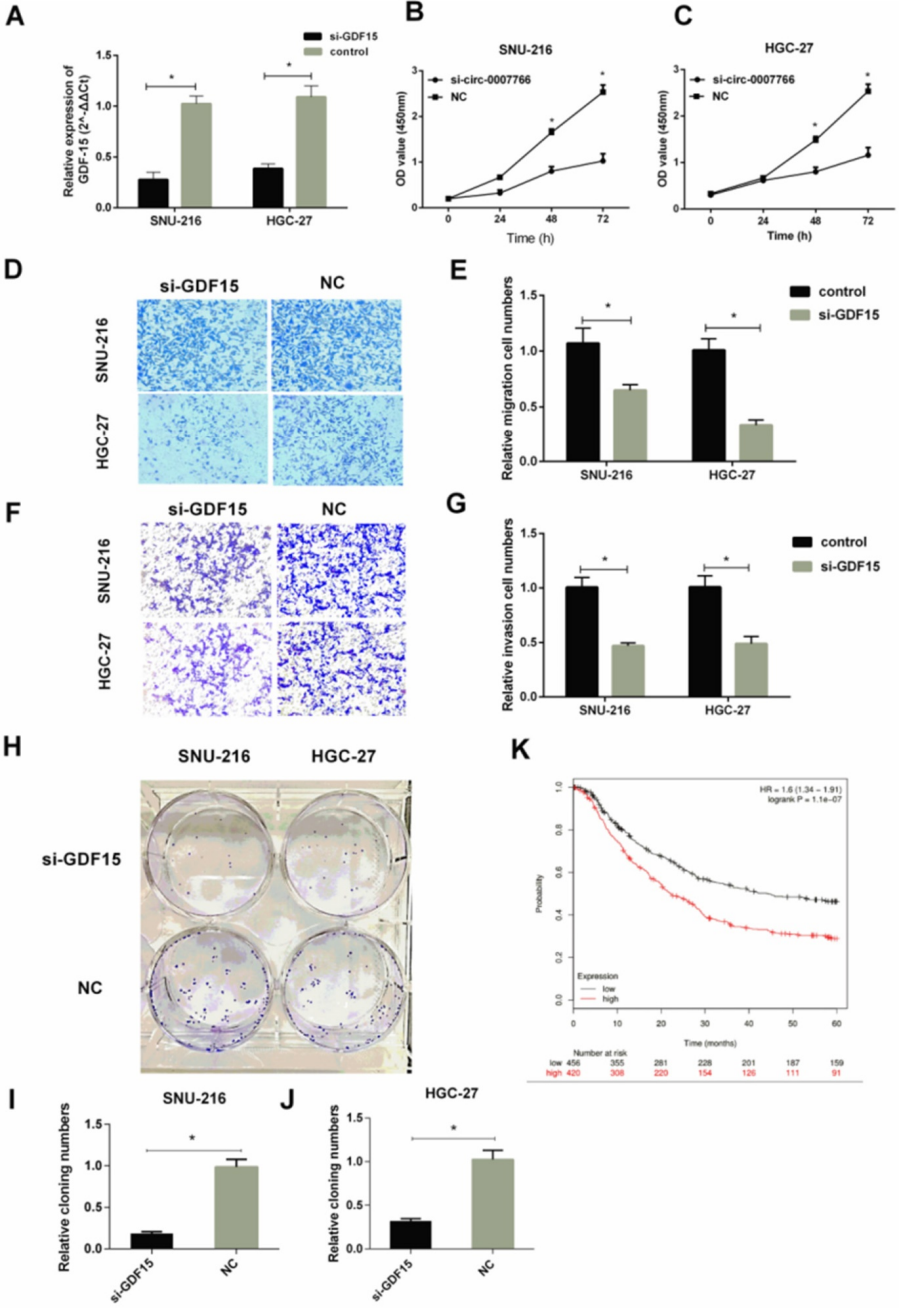
** Silence of *GDF15* inhibited the progression of GC cells *in vitro*. (A)** qRT-PCR analysis verified the effective down-regulation of *GDF15* after the transfection of the siRNA in SNU-216 or HGC-27 cells*.* **(B)** and** (C)** Silence of *GDF15* inhibited cell proliferation as indicated by CCK-8 assays in SNU-216 and HGC-27 cells. **(D-G)** Transwell migration** (D-E)** and invasion assays **(F-G)** were measured, and the results were expressed as relative migration and invasion cell numbers compared with respective control (magnification, x100, *P < 0.05, Student's t-test).** (H-J)** Down-regulation of *GDF15* inhibited the colony formation of SNU-216 **(I)** and HGC-27** (J)** cell lines as demonstrated by colony formation assays. **(K)** Kaplan-Meier plotter analysis of the correlation between *GDF15* expression level and the overall survival of GC patients. (*P < 0.05, Student's t-test).

We transfected GC cell lines SNU-216 and HGC-27 with siRNA for* GDF15*. Then, we assessed the effect of *GDF15* on the proliferation, migration, and invasion of GC cells. qRT-PCR revealed that *GDF15* was significantly down-regulated by* GDF15* siRNA transfection compared with the negative siRNA control (Figure 6A). CCK-8 results showed that *GDF15* knockdown dramatically reduced SNU-216 and HGC-27 cell proliferation compared with control groups (Figure 6B-6C). Knockdown of *GDF15* also reduced the migration (Figure 6D-6E) and invasion (Figure 6F-6G) in SNU-216 and HGC-27 cells compared with cells transfected with the negative control. Next, we applied colony formation assays (Figure 6H) to demonstrate that the down-regulation of *GDF15* inhibited the colony formation of SNU-216 (Figure 6I) and HGC-27 (Figure 6J) cell lines. The correlation between* GDF15* levels and the prognostic outcomes of GC patients was tested by the Kaplan-Meier Plotter website. Patients were stratified into two groups based on an auto select best cut-off. We revealed that *GDF15* expression levels were negatively correlated with survival data (Figure 6K).

### MiR-1233-3p inhibitor (AMO-1233-3p) reversed the down-regulation effect of siRNA for hsa-circ-0007766 (si-circ) in GC cell lines

**Figure 7:**
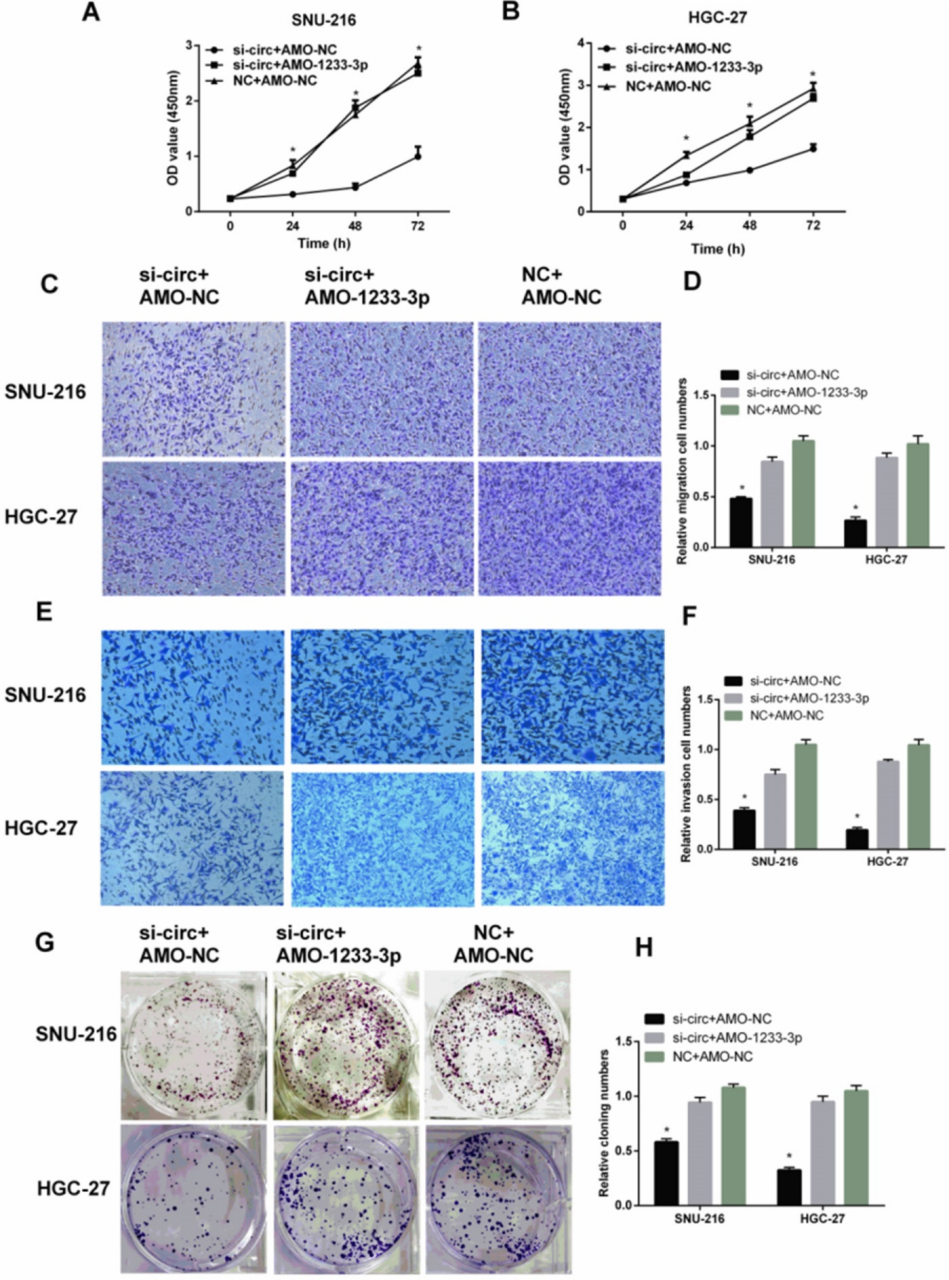
** MiR-1233-3p inhibitor (AMO-1233-3p) reversed the down-regulation effect of siRNA for hsa-circ-0007766 (si-circ) in GC cell lines SNU-216 and HGC-27. (A)** and **(B)** AMO-1233-3p reversed the inhibitory effect of si-circ on cell proliferation by CCK-8 assay. **(C-F)** AMO-1233-3p reversed the inhibitory effect of si-circ on cell migration** (C-D)** and invasion **(E-F)** by transwell assay. **(G-H)** Colony formation assay showed that the si-sirc+AMO-1233-3p group increased in colony numbers of SNU-216 and HGC-27 cell lines compared with the si-circ+AMO-NC group. (*P < 0.05, Student's t-test).

To further verify the interaction between hsa-circ-0007766 and miR-1233-3p, and the binding between miR-1233-3p and *GDF15*, we used siRNA for hsa-circ-0007766 (si-circ) or si-circ plus miR-1233-3p inhibitor (AMO-1233 -3p) to transfect SNU-216 and HGC-27 cells. Subsequently, we used CCK-8 assay (Figure 7A-7B), transwell assay (Figure 7C-7F), and colony formation assay (Figure 7G-7H) to evaluate the effect of cell proliferation, migration, and invasion of GC cell lines SNU-216 and HGC-27. We found that AMO-1233-3p could significantly reverse the down-regulation effect of si-circ in GC cell lines SNU-216 and HGC-27.

### Hsa-circ-0007766 modulated the expression of miR-1233-3p target GDF15

**Figure 8:**
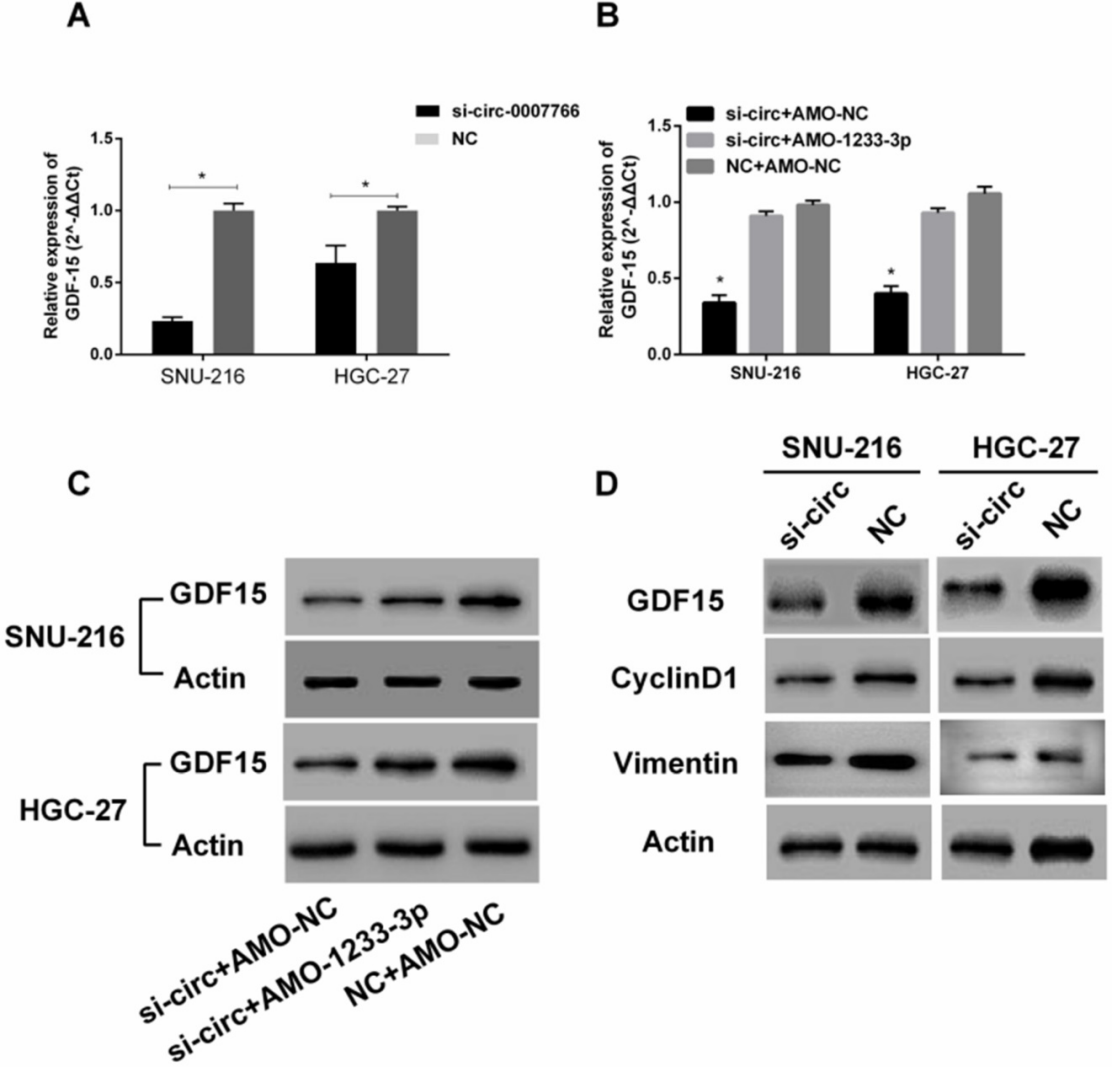
** Hsa-circ-0007766 modulated the expression of miR-1233-3p target *GDF15*. (A)** Transfection of siRNA for hsa-circ-0007766 inhibits the expression of GDF15.** (B)** The* GDF15* mRNA expression levels were reduced by the inhibition of hsa-circ-0007766 and reversed by the miR-1233-3p inhibitor (AMO-1233-3p).** (C)** The protein expression levels of GDF15 were in accordance with its mRNA expression levels. **(D)** Western blot analysis of *GDF15*, cell proliferation-related protein *CyclinD1,* and EMT related protein *Vimentin* in GC cells transfected with siRNA for hsa-circ-0007766 or negative control. (*P < 0.05, Student's t-test).

Subsequently, we investigated whether hsa-circ-0007766 exerted its effect by modulating the expression of *GDF15*. As shown in Figure 8A and 8D, the knockdown of hsa-circ-0007766 could down-regulate the expression of* GDF15* by qRT-PCR or western blot. Besides, we co-transfected si-circ and AMO-1233-3p to detect the expression of *GDF15* further. We found that the AMO-1233-3p could partially rescue the inhibitory effect of si-circ to the expression levels of *GDF15* by qRT-PCR (Figure 8B) and Western blot (Figure 8C). To investigate the underlying mechanisms of si-circ that suppressed cell proliferation, we measured a series of critical proteins related to cell progression by western blot assays. We found that *GDF15*, cell proliferation-related protein *CyclinD1*, and EMT-related protein *Vimentin* were down-regulated after the si-circ transfection compared with the negative control group (Figure 8D).

### ROC analysis was conducted to evaluate the diagnostic value of hsa-circ-0007766 for GC patients

**Figure 9:**
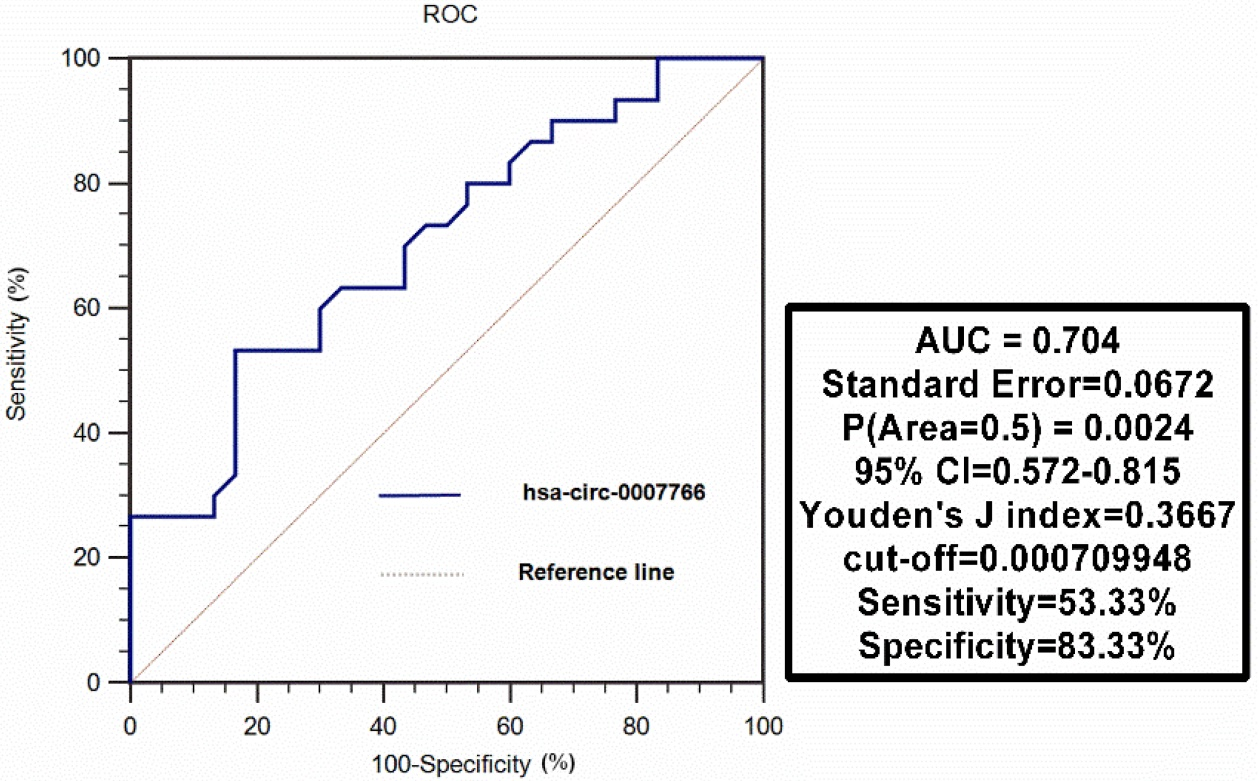
** ROC curve analysis of hsa-circ-0007766 in gastric carcinoma tissues.** Hsa-circ-0007766 was extracted from 30 pairs of gastric carcinoma tissues, and we evaluated the function of hsa-circ-0007766 as a biomarker using ROC analysis. ROC: receiver operating characteristic, AUC: area under curve, CI: confidence interval.

Hsa-circ-0007766 was extracted from 30 pairs of gastric carcinoma tissues, and we differentiated tumors from matched adjacent normal tissues to evaluate the diagnostic value of hsa-circ-0007766 as a biomarker using ROC analysis. We found that the AUC was 0.704 (95% CI, [0.572-0.815]; P = 0.0024), with a cut-off value of 0.000709948 (sensitivity= 53.33%; specificity= 83.33%) (Figure 9).

## Discussion

CircRNAs have been identified as abundant stable ncRNAs with high-throughput sequencing and bioinformatic analysis 10, 30. However, in contrast to the full knowledge of lncRNAs and miRNAs, the function of circRNAs has been rarely elucidated.

Although studies have shown that the sponge function of circRNA is a kind of universal phenomenon 5, 6, 8, 31, some recent publications suggest that circRNAs do not necessarily function as miRNA sponges in human and mouse cells 32, 33. Compared with other competitive endogenous RNAs, circRNAs have more stable miRNA response elements and higher intracellular expression. Meanwhile, circRNAs are predominantly located in the cytoplasm and share the same location with miRNAs. However, a recent study also found circular intronic RNAs (ciRNAs) and EIciRNAs were mainly located in the nucleus and might act as the transcription regulators 34. Furthermore, a few studies reported that some endogenous circRNAs with open reading frames or IRES could translate polypeptides or proteins under certain conditions 7, 13.

In this study, we found that hsa-circ-0007766 was highly expressed in gastric carcinoma tissues and verified its high expression in gastric carcinoma cell lines *in vitro* experiments. We then analyzed the results of qRT-PCR and found that the expression level of hsa-circ-0007766 was significantly correlated with histological grade. Next, we confirmed that convergent and divergent primers could both amplify cDNA of circRNA. However, gDNA could only be amplified by convergent primers. The results of Sanger sequencing verified the back-spliced junction, and the gel electrophoresis confirmed the product of qPCR for circRNA and linear RNA. Subsequently, we applied the technique of fluorescent *in situ* hybridization (FISH) to identify that hsa-circ-0007766 was mainly located in the cytoplasm. What's more, the CCK-8 assays, colony formation assays, transwell migration assays, and transwell invasion assays demonstrated that knockdown of hsa-circ-0007766 could significantly suppress the progression abilities of SNU-216 and HGC-27 cell lines. Taken together, these findings uncovered a remarkable role of hsa-circ-0007766 as an oncogene in GC. Specially, we gradually confirmed the unique role of the hsa-circ-0007766 in the gene regulatory network with pulldown assay and luciferase reporter assay, and the results were consistent with our hypothesis that hsa-circ-0007766 regulates *GDF15* by sponging miR-1233-3p. To our knowledge, this is the first report to illustrate the relation between circRNA and gene *GDF15*.

Bioinformatic analysis was applied to find the binding miRNAs with hsa-circ-0007766 (Figure 1). We searched CircInteractome, Starbase v2.0, Targetscan, and RNAhybrid, miR-1233-3p was finally picked out based on the context+ score percentile and PCR results of miRNAs from the intersection part of these databases 35-38. Although the knockdown of certain circRNAs had been verified ineffective to the expression level of corresponding miRNAs by some studies 5, 8, 31, 39, we consistently discovered the stable and significant up-regulation of miR-1233-3p after the knockdown of hsa-circ-0007766 with siRNA. Then pulldown assay was conducted to confirm this binding effect 40. It should be pointed out that several articles reported that circRNAs could mainly inhibit the activity rather than the transcription level of miRNAs to some extent 5, 39, 41. Subsequently, we adopted the luciferase reporter assay to verify the binding site between miR-1233-3p and the 3'UTR of *GDF15*, which had been reported by Teng et al. 42. We further confirmed the relation between *GDF15* and miR-1233-3p by regulating the miR-1233-3p with miRNA mimics or inhibitor to test the *GDF15* mRNA and protein level via PCR and western-blot, respectively. The expression level of *GDF15* was not significantly changed after the transfection of miR-1233-3p inhibitor. This phenomenon might result from a low abundance of miR-1233-3p and a more prominent binding effect of other miRNAs.

Mir-1233-3p has been reported by several studies and may serve as a new biomarker for cancer diagnosis in the future 41, 43, 44. This study further elaborated on the anti-tumor effect of miR-1233-3p, which was consistent with a previous report 41. Some recent studies revealed that *GDF15*, also known as *MIC-1*, was related to the expression level of the iron regulatory protein hepcidin 45, cancer cachexia 21, and SMAD protein activation in the myocardium 23. What's more, *GDF15* could also act as an oncogene in GC as it might promote the progression of GC by stimulating GC cell invasion via an extracellular signal-regulated kinase-1/2-dependent pathway to up-regulate the uPA activation system 46. Besides, several studies also elaborated on the characteristics of *GDF15* to influence cancer stem-cell-like properties, activate apoptosis via the *CXXC4*-*GDF15* axis, participates in fibroblast activation in GC 47. Above all, *GDF15* was widely accepted as a factor to promote cell viability, invasion, migration, angiogenesis, and apoptosis in human GC cell lines, which was consistent with our present research.

Finally, we conducted the ROC analysis and found that the AUC was 0.704 (95% CI, [0.572-0.815]; p = 0.0024), with a sensitivity of 53.33% and a specificity of 83.33%. The results indicated that the tissue level of hsa-circ-0007766 maybe as a potential diagnostic marker for GC.

In this study, we found that si-circ transfection impeded the expression of *GDF15*. Hence, we speculated that the knockdown of hsa-circ-0007766 could inhibit the progression of GC cell lines via the miR-1233-3p/*GDF15* axis. Further studies are still needed to validate this mechanism. Moreover, accumulated evidence indicates that numerous circRNAs are enriched in the central neural system and not necessarily act as miRNAs sponge in human and mouse cell lines, so it should be further investigated concerning hsa-circ-0007766/miR-1233-3p/*GDF15* axis in GC cell lines as the low abundance of hsa-circ-0007766. Furthermore, this axis may not play the dominant biological role in GC as the complexity of tumor heterogeneity.

In conclusion, our study explicitly elucidated the role of hsa-circ-0007766 in GC for the first time. The results showed that hsa-circ-0007766 was significantly high-expressed in GC and correlated with histological grade in terms of clinical-pathological parameters. Moreover, Down-regulated hsa-circ-0007766 suppressed cell proliferation, migration, and invasion *in vitro*. Subsequently, we explored the potential mechanism, and it suggested that hsa-circ-0007766 influenced the progression of GC cell lines via miR-1233-3p/*GDF15* axis partly. Taken together, hsa-circ-0007766 could serve as a diagnostic and therapeutic biomarker for GC.

## Figures and Tables

**Table 1:** The sequence of primers and probes

Names	Sequence (5'-3')
hsa-circ-0007766-F	TGTGTGACTGCCTGTCCCTG
hsa-circ-0007766-R	AATCCGCAGCCTCTGCAGTG
si-hsa-circ-0007766 (si-circ)	TGCCTGTCCCTGATATCCA
GDF15-F	TGCCTGTCCCTGATATCCA
GDF15-R	GCAGGTCCTCGTAGCGTTTC
U6-F	CTCGCTTCGGCAGCACA
U6-R	AACGCTTCACGAATTTGCGT
hsa-circ-0007766-biotin-probe	ATATCAGGGACAGGCAGTCA
hsa-circ-0007766-scramble-probe	ATGACTGAGTCGCGAAGCAA
gDNA-F	TCACAGAGATCTTGAAAGGAGGG
gDNA-R	GAGAGCGGTTGGTGTCTATCAG
GAPDH-F	ACCCACTCCTCCACCTTTGAC
GAPDH-R	TGTTGCTGTAGCCAAATTCGTT

**Note** F, forward; R, reverse. The sequence information of all siRNA controls, ASO controls, miRNA primers, and Smart Silencer controls is not public according to the declaration of RiboBio (Guangzhou, China).

**Table 2:** Correlations between the expression level of hsa-circ-0007766 and the clinicopathological features in 30 pairs of GC tissues

Characteristics		Case	Circ-0007766 expression		p-value
			Low	High	
All		30	17	13	
Age (years)	<65	11	7	4	0.708
	≥65	19	10	9	
Gender	Male	17	12	5	0.196
	Female	13	12	1	
Histology	Poor-differentiated	15	5	10	0.025*
	well-differentiated	15	12	3	
TNM stage	I-II	5	2	3	0.628
	III-IV	25	15	10	

*p<0.05.

## Abbreviations

ceRNA: competing endogenous RNA
circRNA: circular RNA
EIciRNAs: exon-intron circRNAs
miRNA: microRNA
GC: gastric carcinoma
*GDF15*: growth differentiation factor 15
qRT-PCR: quantitative real-time reverse transcription-polymerase chain reaction
CCK-8 assay: Cell Counting kit-8 assay
ROC: receiver operating characteristic
AUC: area under curve
CI: confidence interval
